# Host population density and presence of predators as key factors influencing the number of gregarious parasitoid *Anaphes flavipes* offspring

**DOI:** 10.1038/s41598-019-42503-4

**Published:** 2019-04-15

**Authors:** Alena Samková, Jiří Hadrava, Jiří Skuhrovec, Petr Janšta

**Affiliations:** 1 Department of Zoology, Faculty of Science, Charles University, Viničná 7, CZ-128 43 Praha 2, Czech Republic; 2 Institute of Entomology, Biological Centre, Czech Academy of Sciences, Branišovská 31, CZ-370 05 České Budějovice, Czech Republic; 3 Crop Research Institute, Drnovská 507, CZ-161 06 Praha 6 – Ruzyně, Czech Republic

## Abstract

The number of parasitoids developed per host is one of the major factors that influences future adult body size and reproductive success. Here, we examined four external factors (host species, heritability, host population density, and presence of predators) that can affect the number of the gregarious parasitoid *Anaphes flavipes* (Förster, 1841) (Hymenoptera: Mymaridae) wasps developing in one host. The effect of host population density on the number of parasitoid offspring developed per host was confirmed, and for the first time, we also showed that the number of offspring per host is influenced by the presence of predators. Low host density and presence of predators increases the number of wasps developed in one host egg. However, a higher number of *A. flavipes* in one host reduces *A. flavipes* body size and hence its future individual fertility and fitness. Our results highlighted the importance of some external factors that distinctly affect the number of wasp offspring. Therefore, in this context, we suggest that in comparison to solitary parasitoids, the gregarious parasitoid *A. flavipes* can better respond to various external factors and can more flexibly change its population density.

## Introduction

Females of solitary parasitoids lay one or more eggs into a single host. If more than one egg is oviposited, then the larvae compete, and only one survives^1^. In contrast, gregarious parasitoids employ a strategy in which one or more parasitoids may develop in one host^2^. This latter strategy evolved independently at least 43 times, probably from the solitary type of wasp^3,4^.

Some parasitoid females can recognize the quality of the host before laying eggs by using the ovipositor and antennae and thus can directly choose the size and/or the sex ratio of the clutch (i.e., planned fertility)^5–8^. In addition, the future body size of a solitary parasitoid positively correlates with the size of the host^9–11^, while the body size of the offspring of gregarious parasitoids is closely related to not only the size of the host but also the number of parasitoids developing in the host^12,13^. One individual parasitoid larva per host usually has a surplus of food; in contrast, more individuals developing in one host may lack food^14,15^. The amount of food for larval development often positively correlates with the adult body size of parasitoids^12,16,17^. According to the “adult size-fitness hypothesis”, which has been supported several times within hymenopteran parasitoids, in comparison to smaller females, larger females have more offspring and hence higher fecundity and fitness^13,18^. Furthermore, the number of parasitoids developing in one host can also be influenced by the suitability of the host (i.e., host size^7,19^ or host age^7^), host population density^20,21^ or sex ratio of offspring^7,12^. The sex ratio of gregarious parasitoids is usually equal between male and female^22^. However, in many cases, mothers produce as few males as possible to allow fertilization of all available females^8,23,24^. As a result, some combinations of the number and sex ratio of offspring are more advantageous than other combinations^8,25^ and favoured by natural selection to maximize individual fitness^8,26^.

The wasp *Anaphes flavipes* (Förster, 1841) (Hymenoptera: Mymaridae), an idiobiont gregarious egg parasitoid, is a suitable model to study this decision-making process. Although females of *A. flavipes* mostly oviposit two or three offspring into a single host, they can lay one to seven offspring into a single host egg, with 35 possible sex ratios^25^. The host spectrum of the wasp includes species of the genera *Lema* and *Oulema* (Coleoptera: Chrysomelidae), including an economically important crop pest - the cereal leaf beetle *O. melanopus*^25,27^. Recently, we observed that in comparison to smaller females, larger females have more offspring and that the adult size of the offspring is directly determined by the number of offspring developing in one host egg^28^.

In this study, we focused on testing potential factors that may influence the number of offspring developing in one host using *A. flavipes* as a model. We tested whether the number of offspring per host changed under the influence of the following factors: (1) *Host species*. In general, females influence the number of offspring developing per host according to the host species, and the size of the host positively influences the size of parasitoid offspring^7,19^. However, in some cases, larger hosts do not indicate higher nutritional value^29,30^, and parasitoids from smaller but more nutritionally valuable hosts have a larger body size^31^. Eggs of the *Oulema* species are the same size^28^. Therefore, we expect that species of *Oulema* do not differ in nutritional value and do not affect either host preference or the number of offspring. (2) *Heritability*. We hypothesized that the number of offspring developing in one host could be “passed on” from mother to daughter, and daughters inherit a mother´s strategy and lay a similar number of offspring into one host. (3) *Host population density*. Host density influences the number of parasitoids developed in one host^20,21^. We assume that with a plethora of host eggs available, the female *A. flavipes* also increases her fitness if she lays a smaller number of offspring into one host, as this would ensure a larger body size of the offspring^28^. (4) *Presence of predators*. Intraguild predation (IGP), a herbivore-parasitoid-predator relationship in which the parasitized herbivore is also the predator’s prey^32^, causes changes in the reproductive behaviour of a parasitoid, e.g., a parasitoid may not lay eggs into the host in the presence of the predator^33^.

## Results

### Host species

Host species (*O. duftschmidi* (Redtenbacher, 1874), *O. gallaeciana* (Heyden, 1879) and *O. melanopus* (Linnaeus, 1758)) do not affect the reproduction of wasps, i.e., the number of offspring developed in one host egg (p = 0.207, F = 1.584, df = 361, n = 354, ANOVA).

### Heritability

The mother and offspring showed no significant similarity in the number of offspring developed in one host egg (p > 0.0881, slope = 0.021, sd = 0.1396, t = 0.15, n = 36, linear regression).

### Host population density

**Figure 1:**
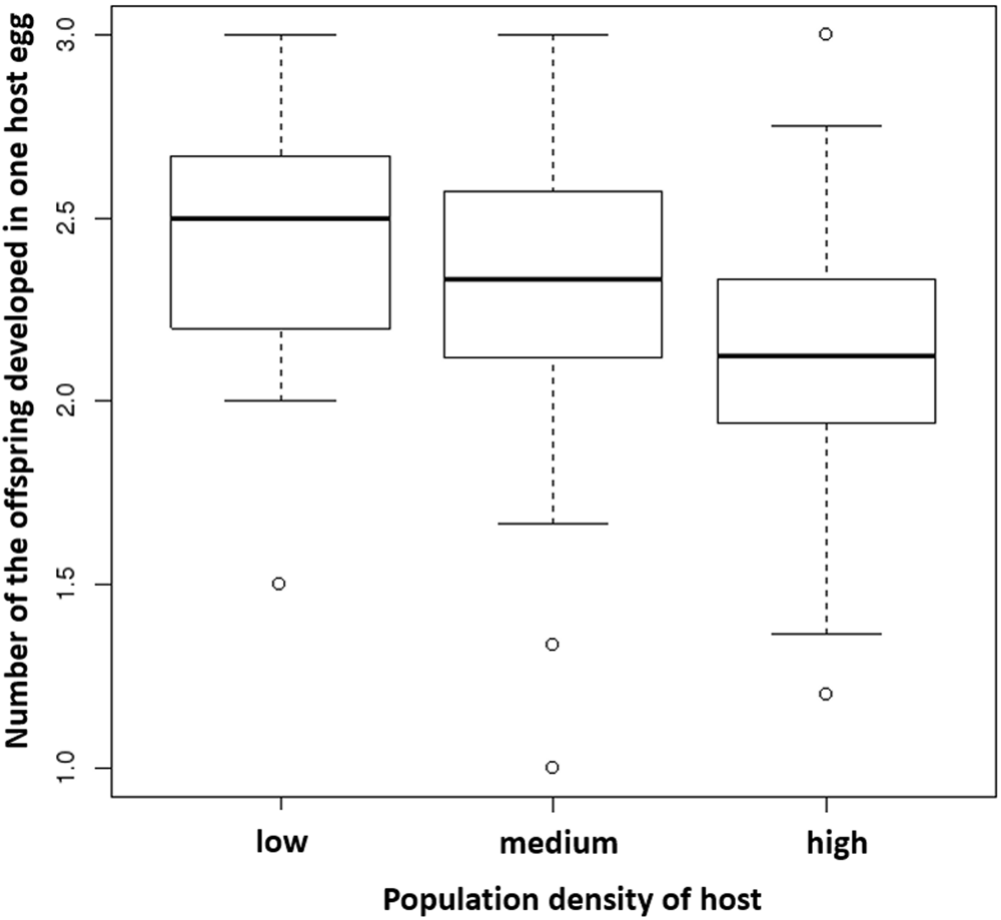
The relationship between the number of offspring developed in one host egg and the population density of the host.

**Figure 2:**
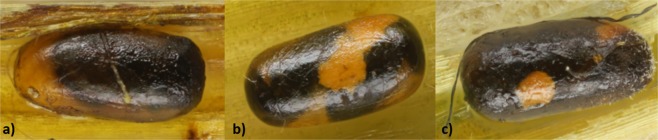
The adult body sizes of wasps decline as offspring number per host increases. One (**a**), two (**b**) and three (**c**) wasps developing per host egg.

**Figure 3:**
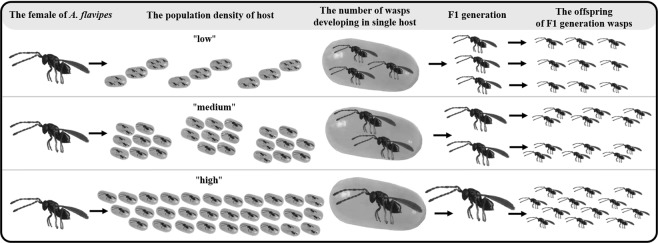
The number of wasps developing in one host egg, their adult body size and future fertility are influenced by the host population density of the host. Each female has 34 offspring, but the size of their offspring depends on the number of individuals developed in one host^28^. Wasps lay a higher number of eggs into one host egg and therefore produce smaller offspring if the population density of hosts is “low” compared “medium” and “high” population densities of hosts. The body size of the F1 generation female wasps determines the number of developed offspring. The number of offspring per host egg is illustrative because it does not reflect the real number of developed offspring.

The number of offspring developed in one host egg depends on the population density of the host. At a higher population density of the host, the females parasitized more host eggs (p = 0.000586, R-Sq = 0.114, df = 123, F = 7.911, ANOVA), but on average, the number of developed offspring from one host egg was lower (p = 0.000597, R-Sq = 0.114 df = 123 F = 7.8914.52, ANOVA). The total number of offspring developed by one female increased only slightly but still significantly (p = 0.0196, R-Sq = 0.0619, df = 123, F = 4.058, ANOVA). On average, at low, medium, and high population densities of the host, the wasps laid 2.5, 2.25, and 2.1 offspring per host, respectively (Figs 1, 2, 3).

### Presence of predator

**Figure 4:**
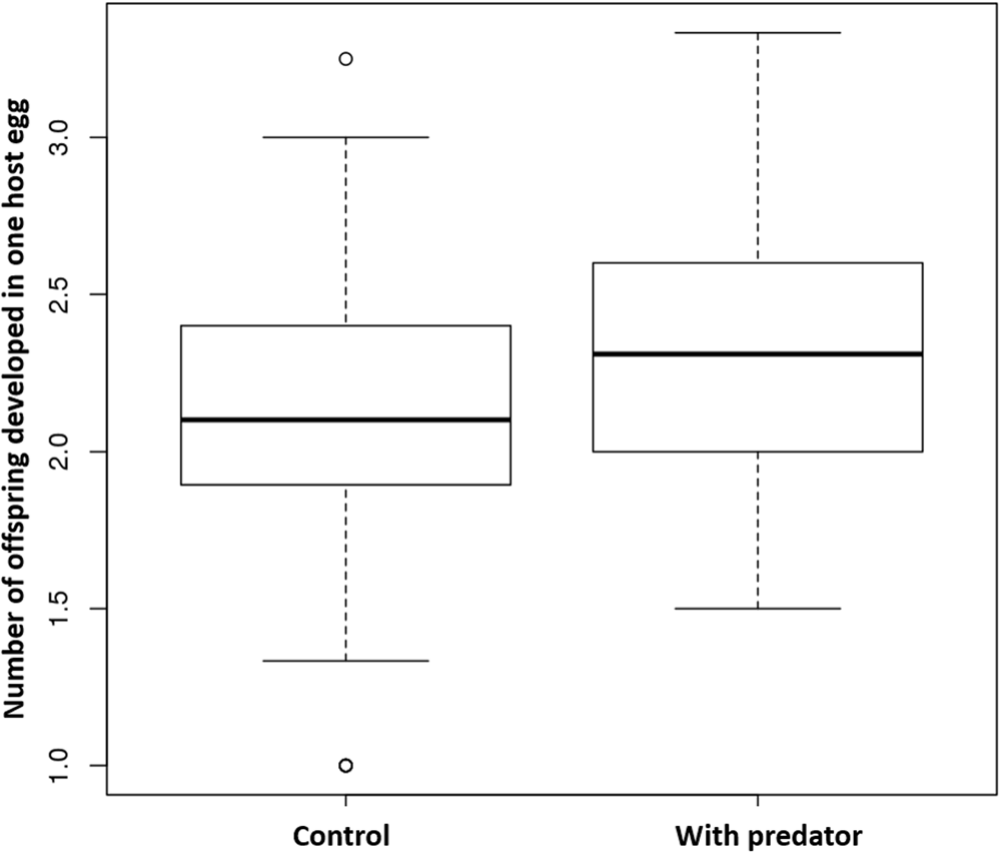
The number of offspring developed in one host egg in the experiment without the presence of a predator (Control) vs. the experiment with a predator (With predator).

In the presence of a predator, the wasps laid 0.21 eggs more into one host egg than the number of eggs from wasps without a predator (p = 0.00038, slope = 0.21244, sd = 0.05913, t = 3.592, n = 297, linear regression, Fig. 4). However, the total number of parasitized host eggs (p = 0.871, n = 297) and the total number of developed offspring by one female did not differ significantly from the scenario without a predator (p = 0.339, n = 297).

## Discussion

Our results confirmed that the parasitic wasp *A. flavipes* lays a lower number of offspring into each host egg at higher population densities of the host than lower population densities. These results correspond to the results of several studies that described a special strategy of female parasitic wasps called “planned fertility”. Planned fertility has been documented for the females of *Mastrus ridibundus* (Gravenhorst, 1829) (Hym.: Ichneumonoidea)^20^ and *Hyssopus pallidus* (Askew, 1964) (Hym.: Chalcidoidea)^21^, where the number of offspring developed in one host depends on the availability of the host. The females of *Nasonia vitripennis* (Walker, 1836)^7^ and *Trichogramma embryophagum* (Hartig, 1838)^19^ (both Chalcidoidea) recognize the size of hosts and accordingly lay a certain number of eggs into a host. These results confirmed that the number of *N. vitripennis* and *T. embryophagum* eggs laid into a single host is positively correlated with host size. These cases also showed that the amount of food available for the development of one specimen within different hosts is not necessarily a limiting factor^13,19^.

Our previous study on *A. flavipes* showed that its hosts, the three *Oulema* species, do not differ in size^28^. Consequently, the amount of food sources in the host in this case is constant and therefore likely limiting parasitoid larval development. While a single wasp developing in a single host egg enjoys a nutritional surplus, the food sources may be insufficient for more wasps developing in a single host egg (Figs 2, 3). As the number of *A. flavipes* offspring developing in one host increases, their body size (and therefore number of offspring F1 generation) decreases. Previously, we have shown that the number of offspring developed in one host is the major factor affecting the adult body size of *A. flavipes*^28^. Our current results highlight that the number of offspring developed in one host egg is affected by the population density of the host.

The host beetles of *A. flavipes* (*Oulema duftschmidi, O. gallaeciana*, and *O. melanopus*) often have high population densities, especially in agroecosystems, as economically significant important pests of cereals^34,35^. In those environments, a high population density of the host means that the parasitic wasps might lay a comparatively lower number of offspring into single host eggs. As a result, the emerging larger adult wasps may exhibit a higher fitness and therefore reach higher population densities in a shorter amount of time. Large numbers of parasitoids can more effectively regulate host populations, and over time, host populations will decrease due to high parasitism. In contrast, low population densities of a host would result in a higher number of wasp offspring in one host egg, decreasing parasitoid overall fitness. Thus, the population density of the host helps to keep a parasitoid-host relationship in equilibrium, and host population density depends on the population density and the number of adult wasps developed in one host egg.

Intraguild predation (IGP) is a trophic interaction between organisms sharing the same resource (e.g., herbivore-parasitoid-predator) and can be influenced by protagonist host specificity, size, mobility, aggressiveness, extraguild prey density^36^ and parasitism^37^. Parasitoids are usually intraguild prey because the parasitized hosts are a potential prey for predators^32,33^. For parasitoids, it is advantageous to avoid places with predators to reduce the risk of predation of their offspring^38^.

In our experiments, we used *Coccinella septempunctata* Linnaeus, 1758 (Coleoptera: Coccinellidae) as the predator because they eat the eggs of *Oulema*^39,40^ and leave a chemical trail^41^. Avoidance of parasitization in the presence of the predator *C. septempunctata* has been observed in several solitary parasitoids, e.g., *Aphidius ervi* Haliday, 1834^33,38^, *A. eadyi* Starý, Gonzales & Hall, 1980 and *Praon volucre* Haliday, 1833^38^. The parasitoid *A. ervi* also recognized the chemical traces of *Harmonia axyridis*^42^. Since our model species, *A. flavipes*, is a gregarious parasitoid, in addition to the number of parasitized host eggs and the total number of developed offspring of one female, we also measured whether the number of offspring developed in one host egg was affected by the presence of a predator.

In our experiment, the total number of developed offspring and the number of parasitized host eggs by one female did not correlate with the presence or absence of a predator. Wasps parasitizing host eggs regardless of the presence of a predator could have been related to their full egg load (no prior laying experience). Correspondingly, others have found^33^ that wasps that had already laid eggs (i.e., had a lower egg load) tended to avoid parasitization in places with a predator. However, the presence of a predator affected the number of offspring per host. In one host egg, females laid a higher number of offspring in the presence of a predator rather than in its absence. Adults of predator *Harmonia axyridis* (Pallas, 1773) prefer nonparasitized hosts over parasitized ones as food^37^. Thus, we assumed that wasps demonstrate this preference to ensure the survival of more offspring if a predator avoids the particular parasitized host egg.

Snyder & Ives^43^ have shown that the adults of *H. axyridis* did not prefer hosts parasitized by the braconid *Aphidius ervi*; thus, the pest population was reduced by both a predator and parasitoid, which streamlined the biological control of the pest. In contrast, larvae of *H. axyridis* do not distinguish between nonparasitized and parasitized hosts, and the IPG reduces the population of the parasitoid as a consequence of predator pressure^37,44^. In the case of *A. flavipes*, in the presence of the predator, individual offspring fitness was reduced due to females laying more offspring in one host egg, resulting in smaller and less fertile offspring^28^; however, overall brood fitness decreased as predators preferred nonparasitized host eggs.

Two major factors are known to determine the reproductive success of parasitoids: (1) the number of available hosts (host density) and (2) the number of parasitoid mature eggs^11^. At high densities of a host, a wasp lays a smaller number of eggs in one host. Thus, if fewer individuals develop in a host egg, then they will have a relatively larger body size^45^, which then positively correlates with the number of their offspring (mature eggs)^46,47^. A similar pattern has been reported in the gregarious wasp *Anagrus* spp.^48^, where the number of mature eggs increased with a higher population density of the host. Different numbers of offspring developing in one host can respond to the changing population density of the host by varying body size and thereby future reproductive success. In addition, the number of offspring developed in one host also depends on the presence of a predator because parasitoid females load more offspring into one host. However, in all experiments, we measured the number of hatched offspring from one host egg and not the number of laid eggs (same as in the previous study^49^), which do not reflect the mortality rate.

Overall, our results demonstrate the importance of the relationship of body size and fertility with the number of offspring developed per host for the gregarious parasitoid *A. flavipes* and responses to external factors. Similar to the results of previous studies^20,21^, we confirmed that these external factors are different population densities of the host and, as a new factor, the presence of the predator. Our study of *A. flavipes* thus shows that external factors affect the individual offspring fitness of parasitoids and may enhance the effectiveness of biological control.

## Material and Methods

### Parasitic wasps

Parasitic wasps, *A. flavipes*, were individually collected from host eggs (*Oulema* spp.) from the end of April until the end of June 2014–2016 in cereal fields (barley and wheat) in two localities in Prague-Suchdol, Czech Republic (GPS: 50.1385422 N, 14.3695547E; 50.1367269 N, 14.3638039E). The parasitized host eggs were stored in Petri dishes with moistened filter paper until adult wasps emerged. These “wild” wasps were used as an initial population to rear the next generations of parasitoids in an environmental chamber at 22 ± 2 °C, a relative humidity of 40–60% and 24 hours light. All “next generation” females of *A. flavipes* were allowed to mate and entered the experiments no more than 24 hours post emergence. Each mated female was placed in a Petri dish with host eggs. Before the start of the experience and during the experiment, all females were not fed, and they had constant access to water.

### Host species

The host species of the genus *Oulema* (*O. duftschmidi*, *O. gallaeciana* and *O. melanopus*) were established from the adults collected at the same time as host eggs in three localities in the Czech Republic (two in Prague-Suchdol (identical to those for the parasitic wasps) and Police nad Metují (GPS: 50.5277906 N, 16.2456192E)) using a net or individual collection. The host *Oulema* species were determined to the species level: (1) *O. gallaeciana* (*Og*), (2) *O. duftschmidi* (*Od*), and (3) *O. melanopus* (*Om*). Distinguishing between *Od* and *Om* was determined based on the morphology of genitals^34^. In the following experiments, all eggs of *Oulema* species were used without exact identification on the species due to the results of the host species. The *Oulema* beetles were bred in Petri dishes (diameter 8.5 cm, for pairs of hosts) or plastic boxes (10 × 10 × 5.5 cm or 20 × 20 × 18 for more individuals) with moistened filter paper. Adults were fed grain leaves and had unlimited access to water. The *Oulema* species laid their eggs on cereal leaves in an environmental chamber at 22 ± 2 °C, a relative humidity of 40–60% and 24 hours light. The host eggs were removed on a 1 cm long piece of leaf and then used in the experiment. Wasps refused host eggs older than 72 hours because the emerging larvae of beetles can have sclerotised mandibles, which could damage the eggs of the parasitoids^25^. In our experiments, we used eggs not older than 24 hours.

### Laboratory experiments

All laboratory experiments were performed in Petri dishes (diameter 8.5 cm) in a thermal cabinet at 22 ± 2 °C, relative humidity of 40–60% and 24 hours light. Individual host eggs were removed on the 9th or 10th day after parasitization into 1.5 ml Eppendorf tubes and stored at the same temperature in a thermal cabinet. For each infested host egg, the number of hatched wasps was measured. After the experiment, all wasps were stored in 96% ethanol.

### Experimental design

#### Host species

Twelve host eggs of three different species of the genus *Oulema* (4 eggs of *Og* + 8 eggs of *Od* and *Om*) were offered to each of the 120 female wasps for 8 hours. A total of 354 out of 1440 host eggs were parasitized by a wasp. For each parasitized egg, we counted the number of offspring (developed adults).

#### Heritability

Each female (n = 36) had 12 host eggs available for parasitization for 8 hours in Petri dishes. The same number of host eggs (12) was offered for parasitization (for 8 hours) for one randomly chosen offspring from each of the 36 mothers. The number of developed offspring in each host egg of the mother and her offspring was measured.

#### Host’s population density

**Figure 5:**
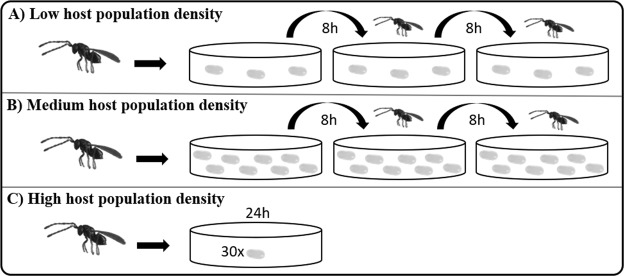
Low (**A**), medium (**B**) and high (**C**) host population densities. A low host density is simulated as 9 host eggs per parasitoid female (3 × 3 host eggs for 8 hours), medium host density is simulated as 24 host eggs per parasitoid female (3 × 8 host eggs for 8 hours), and high host density is simulated as 30 host eggs per parasitoid female for 24 hours.

The different population density of the host was simulated in the laboratory using three different treatments (Fig. 5). In the first treatment, as a simulation of low host population density, each wasp (n = 37) was given 9 host eggs (Fig. 5A) for parasitization (three host eggs were offered for a period of 8 hours). In the second treatment (medium population density), each wasp (n = 44) was given 24 host eggs (Fig. 5B) for parasitization (eight host eggs were offered for a period of 8 hours). In the third treatment (high population density), 30 host eggs (Fig. 5C) were offered for each wasp (n = 46) for 24 hours. Anderson & Paschke^25^ listed the maximum number of offspring of each wasp as 20, so 30 host eggs were used as the optimal simulation of a high population density of hosts. The number of developed offspring in each egg was measured.

#### Presence of predator

The effect of predation on host eggs was simulated by the presence of an adult seven-spot ladybird, *Coccinella septempunctata*. The predator was placed in a Petri dish (8.5 cm) with moistened filter paper for 2 hours without a wasp and without any host eggs to allow the predator leave chemical trails. After 2 hours, the predator was placed in a 1.5 ml Eppendorf tube closed by mesh (100 μm mesh size), and the tube was placed back into the Petri dish. The mated female of *A. flavipes* (n = 78) and 12 eggs of *Oulema* sp. were released for 8 hours in the Petri dish with the predator from the Eppendorf tube. For each female, we measured the total number of offspring per host egg (the number of developed offspring) and the total number of parasitized host eggs. In the control, the wasp (n = 219) received 12 host eggs for parasitization for 8 hours without a predator.

### Statistical data processing

Software R version 3.3.3 (R Core Team 2017)^50^ was used for all statistical analyses.

The effect of host species on the number of offspring developing in one host in the experiment *Host species specificity* was tested with ANOVA. Assumptions of the ANOVA models were verified. Shannon’s entropy was used as a quantitative measure of the evenness of a distribution of offspring among host eggs. Shannon’s entropy was calculated based on the distribution of the number of offspring in the host eggs for each mother and one randomly selected daughter of the mother. The heritability of the number of offspring hatched from one host was tested by linear regression as the effect of the mother’s Shannon’s entropy on daughter’s Shannon’s entropy.

In the analysis *Host’s population density*, treatments were coded as ordered factors, and their effect on a number of offspring in each egg was analysed with ANOVA.

The experiment *Presence of predator* on a number of offspring in each egg was tested with ANOVA.

All analysed data in this study are available in the Supplementary Information.

## Supplementary information

Dataset 1

Dataset 2

Dataset 3

Dataset 4
